# Impact of 5‐Aminosalicylic acid discontinuation in children with ulcerative colitis on biologic therapy: A propensity score‐matched study

**DOI:** 10.1002/jpn3.70415

**Published:** 2026-03-26

**Authors:** Giulia D'Arcangelo, Luca Scarallo, Giulia Mancuso, Mara Corpino, Claudio Romano, Lorenzo Norsa, Serena Arrigo, Matteo Bramuzzo, Maria Teresa Fioretti, Giovanna Zuin, Maria Teresa Illiceto, Paolo Lionetti, Marina Aloi

**Affiliations:** ^1^ Gastroenterology, Hepatology and Cystic Fibrosis Unit, Fondazione IRCCS Cà Granda Ospedale Maggiore Policlinico di Milano Milan Italy; ^2^ Gastroenterology and Nutrition Unit Meyer Children's Hospital IRCCS Florence Italy; ^3^ Department of NEUROFARBA, Meyer Children's Hospital University of Florence Florence Italy; ^4^ Paediatric Gastroenterology and Liver Unit Sapienza University of Rome Rome Italy; ^5^ Paediatric Gastroenterology Ospedale Paediatrico Microcitemico A. Cao, ARNAS Brotzu Cagliari Italy; ^6^ Department of Human Pathology in Adulthood and Childhood “G. Barresi”, Paediatric Gastroenterology Unit University of Messina Messina Italy; ^7^ Vittore Buzzi Children's Hospital University of Milan Milan Italy; ^8^ Department of Paediatrics, Paediatric Hepatology, Gastroenterology and Transplantation ASST Papa Giovanni XXIII Bergamo Italy; ^9^ Gastroenterology and Digestive Endoscopy Unit IRCCS Istituto Giannina Gaslini Genoa Italy; ^10^ Institute for Maternal and Health IRCCS “Burlo Garofolo” Trieste Italy; ^11^ Department of Translational Medical Science, Section of Paediatrics University of Naples “Federico II” Naples Italy; ^12^ Paediatrics, Fondazione IRCCS San Gerardo dei Tintori Monza Italy; ^13^ Paediatric Gastroenterology and Endoscopy Unit, S Spirito Hospital Pescara Italy; ^14^ Department of Pathophysiology and Transplantation Università degli Studi di Milano Milan Italy

**Keywords:** anti‐TNF, mesalamine, outcome, treatment discontinuation

## Abstract

**Objectives:**

5‐Aminosalicylic acid (5‐ASA) is recommended as a first‐line medication in mild to moderate ulcerative colitis (UC), but indications regarding its use in children with moderate to severe disease treated with biologics are lacking. We aimed to evaluate the impact of discontinuing 5‐ASA in children with UC treated with anti‐tumor necrosis factor (anti‐TNF).

**Methods:**

Retrospective, multicenter, case–control study of the Italian Society of Pediatric Gastroenterology, Hepatology and Nutrition inflammatory bowel disease (IBD) study group. Children with UC starting anti‐TNF therapy between January 2018 and January 2023 and with a minimum follow of 6 months were included. Those who discontinued 5‐ASA (cases) were compared to those who continued mesalamine (controls). A Propensity Score analysis was used to match 1:2 with patients by baseline clinical and demographic characteristics. Every 6 months, during a 2‐year follow‐up, data on disease flares, IBD‐related hospitalization, surgery, need for step‐up treatment and acute severe colitis were recorded.

**Results:**

Data from 298 children were collected (92 [31%] cases and 206 [69%] controls) and 227 were included in the final analysis after matching (85 [37.5%] cases and 142 [62.5%] controls]. Children who discontinued 5‐ASA were at higher risk of courses of steroids (Log‐Rank *p* = 0.003) and hospitalization (*p* = 0.08). At the multivariate Cox regression analysis, 5‐ASA discontinuation was identified as an independent predictor of courses of steroids (*p* = 0.003) and hospitalization (*p* = 0.08).

**Conclusions:**

5‐ASA discontinuation might negatively impact the clinical course of children with UC treated with anti‐TNF. Considering the good safety profile of the drug, continuing mesalamine can be suggested as a prudent approach.

## Abstract (highlights)

**What is Known:**

- 5‐Aminosalicylic acid (5‐ASA) is the cornerstone of initial management for mild pediatric ulcerative colitis (UC) and is typically used as an adjunct to systemic corticosteroids in moderate disease.
- Its benefit when initiating biologic therapy remains unclear.
- Current guidelines provide no recommendations on whether to continue 5‐ASA with biologics.

**What is New:**

- Discontinuing 5‐ASA at anti‐tumor necrosis factor (anti‐TNF) initiation increases the risk of corticosteroid use and hospitalization.
- Continuing 5‐ASA alongside anti‐TNF therapy may improve clinical outcomes.
- Given 5‐ASA's favorable safety profile, low cost, and potential to lower relapse risk, continuing it alongside anti‐TNF therapy in children may improve outcomes.

## Graphical abstract

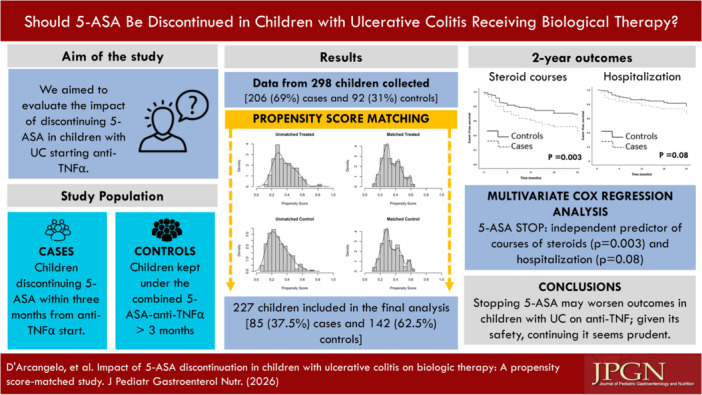

## 1 INTRODUCTION

The European Crohn's and Colitis Organization (ECCO) and European Society for Pediatric Gastroenterology, Hepatology and Nutrition (ESPGHAN) guidelines support a step‐up approach in pediatric ulcerative colitis (UC), with oral and/or rectal 5‐aminosalicylic acid (5‐ASA) as first‐line induction and maintenance for mild disease, while systemic corticosteroids are typically used for induction in moderate‐to‐severe disease or when there is inadequate response to optimized 5‐ASA.^1^, ^2^ Nevertheless, up to 37% of patients on oral 5‐ASA relapse by 12 months^3^ and require therapy escalation to maintain disease remission. Notably, an increased use of anti‐tumor necrosis factor (anti‐TNF) therapies has been reported worldwide in children as a rescue option for 5‐ASA failures.^4^, ^5^

The benefit of continuing or stopping 5‐ASA in pediatric patients who start biologic therapy is currently unknown. The last ECCO/ESPGHAN^2^ guidelines do not provide indications on the efficacy of concomitant intake of 5‐ASA, and no specific study has been conducted, while a few adult studies reported no benefit of adding 5‐ASA to biological or immunomodulator therapy.^6^, ^7^ Despite that, more than 80% of patients entering clinical trials of immunosuppressants, biologics, or oral small molecules continue to use aminosalicylates.^8^ These figures highlight that this topic still needs to be clarified.

In this study, we aimed to define the long‐term outcomes of children treated with biologic therapy, who stopped 5‐ASA within the first 3 months after the initiation of the anti‐TNF and compared them with patients who continued 5‐ASA in combination with anti‐TNF therapy.

## 2 METHODS

### 2.1 Ethics statement

The study was conducted in compliance with ethical standards, adhering to the Declaration of Helsinki's principles. Approval was obtained from the Independent Review Board of the Umberto I Hospital Rome (coordinating center). Confidentiality and privacy of participants' personal information were rigorously maintained throughout the study, with data anonymized in accordance with established ethical guidelines.

### 2.2 Study design and inclusion criteria

This was a multicenter, retrospective, case–control study conducted within nine affiliated centres of the inflammatory bowel disease (IBD) working group of the Italian Society of Pediatric Gastroenterology, Hepatology and Nutrition.

Following an internal call to participate, data from children diagnosed with UC according to the Porto Criteria^9^ and who had started anti‐TNF treatment while under concomitant therapy with 5‐ASA (including mesalamine and sulfasalazine) between October 2013 and October 2022 and with a minimum follow‐up of 6 months from the anti‐TNF initiation were included in the study.

Patients were then divided into cases and controls: patients who discontinued 5‐ASA within 3 months from the biological therapy initiation were considered cases while those kept under the combined 5‐ASA‐anti‐TNF treatment for more than 3 months from the index date were deemed as controls. Children had to be under stable treatment with 5‐ASA for at least 90 days prior to initiation of biologic therapy and had to be kept under biologic for at least 90 days to be included.

Exclusion criteria were intermittent usage of 5‐ASA, exclusively rectal administration of 5‐ASA and history of colectomy before anti TNF initiation.

### 2.3 Data collection

The following clinical data were collected at baseline (date of first anti‐TNF administration, index date): demographic (age, gender) and clinical characteristics, including disease duration, location and disease behavior according to Paris classification,^10^ therapy at diagnosis, and therapies prior to anti‐ TNF, clinical disease activity assessed through the Pediatric Ulcerative Colitis Activity Index (PUCAI),^11^ auxological parameters [height, weight, and Body Mass Index (BMI)]. Laboratory parameters collected at the index date included: blood inflammatory markers (erythrocyte sedimentation rate, [ESR] [mm/h], C‐reactive protein, [CRP] [mg/L], albumin [g/L], and fecal calprotectin [FC] [μg/g]). Severity of the disease at the index date was captured by collecting the following information: prior corticosteroid use (remote defined as 365–90 days prior to anti‐TNF therapy or recent defined as within 90 days prior to anti‐TNF therapy), number of hospitalizations in the year before starting anti‐TNF (categorized as 0, 1–2, or more than 2), episodes of acute severe colitis (ASC) prior to anti TNF initiation, PUCAI after induction (Week 14) in order to determine the presence of clinical remission (PUCAI < 10).

At 6 months and subsequently at 12, 18, and 24 months number and type of each of the following major clinical events were collected: (1) need for systemic corticosteroids (CS), (2) unplanned hospitalization due to exacerbation of UC, clinical relapse (PUCAI > 10 in patients who have previously achieved clinical remission or defined, or an increase of 20 points from the previous assessment), (3) need for surgery, (4) ASC episodes defined as a PUCAI > 65, and (5) need for treatment escalation, defined as the need for additional medical therapy based on lack of sustained response/remission with the use of the previous maintenance agent. When available, data on mucosal healing (MH) (Mayo 0 of FC < 100 μg/g) were collected.

### 2.4 Study outcomes

The primary outcome was to compare the risk of a poor outcome (defined through each of the major events) in cases and controls. The secondary outcome included the identification of factors associated with the occurrence of all the abovementioned clinical events.

### 2.5 Propensity‐score matching analysis

A propensity‐score matching analysis was performed to establish comparable groups of cases and controls for primary outcome analysis.^12^ The following patient and disease‐related baseline variables considered most clinically relevant and consistently available across the cohort were determined a priori and included in the propensity score model predicting the probability of 5‐ASA discontinuation: date of infliximab initiation, recent (< 90 days CS use), ASC at anti‐TNF initiation, disease extension, concomitant immunosuppressive therapy, gender, and clinical remission after induction. Patients discontinuing 5‐ASA were matched 1:2 to patients continuing mesalamine. Patients were matched using a nearest neighbor matching algorithm with no replacement (MatchIt package). The maximum accepted difference between propensity scores in each pair was set at 0.1 standard deviation. Patients in the case and control groups without matching points in the other group within 0.1 standard deviation were discarded. Comparability of baseline characteristics between matched groups was verified using chi‐square test for categorical variables and Mann–Whitney *U* test for continuous variables.

### 2.6 Statistical analysis

Descriptive statistics were presented as median (interquartile range [IQR]) for continuous variables and frequencies (%) for categorical variables. Univariable comparisons were performed using chi‐squared or Fisher's exact tests for categorical variables and a Wilcoxon rank‐sum test for continuous variables. Survival analyses were performed to compare the time‐to‐event outcomes between the sarcopenic and non‐sarcopenic patients. Kaplan–Meier survival curves were generated, and differences between the groups were assessed using the log‐rank test. Hazard ratios (HRs) and their corresponding 95% confidence intervals (CIs) were estimated using Cox proportional hazards regression to account for potential confounders. Multivariable models were constructed by selecting all the covariates that showed a statistically significant association at univariate analysis (*p* < 0.01) and those deemed clinically significant in influencing the outcome of interest, fitted using a backward elimination procedure. Patients were censored at their last recorded follow‐up visit, and no imputation was performed for missing outcome data. All statistical analyses were two‐tailed with the threshold of significance set at *p* < 0.05. Data were analyzed using GraphPad Prism (v.6.0) for Windows, GraphPad software (San Diego, CA, USA) and SPSS (v.23.0) software (SPSS, Chicago, IL, USA).

## 3 RESULTS

Data from 298 children were collected 92 (31%) discontinued 5‐ASA (cases) and 206 (69%) maintained 5‐ASA (controls). The characteristics of the unmatched cohort are displayed in Supporting Information: Table 1.

**Figure 1:**
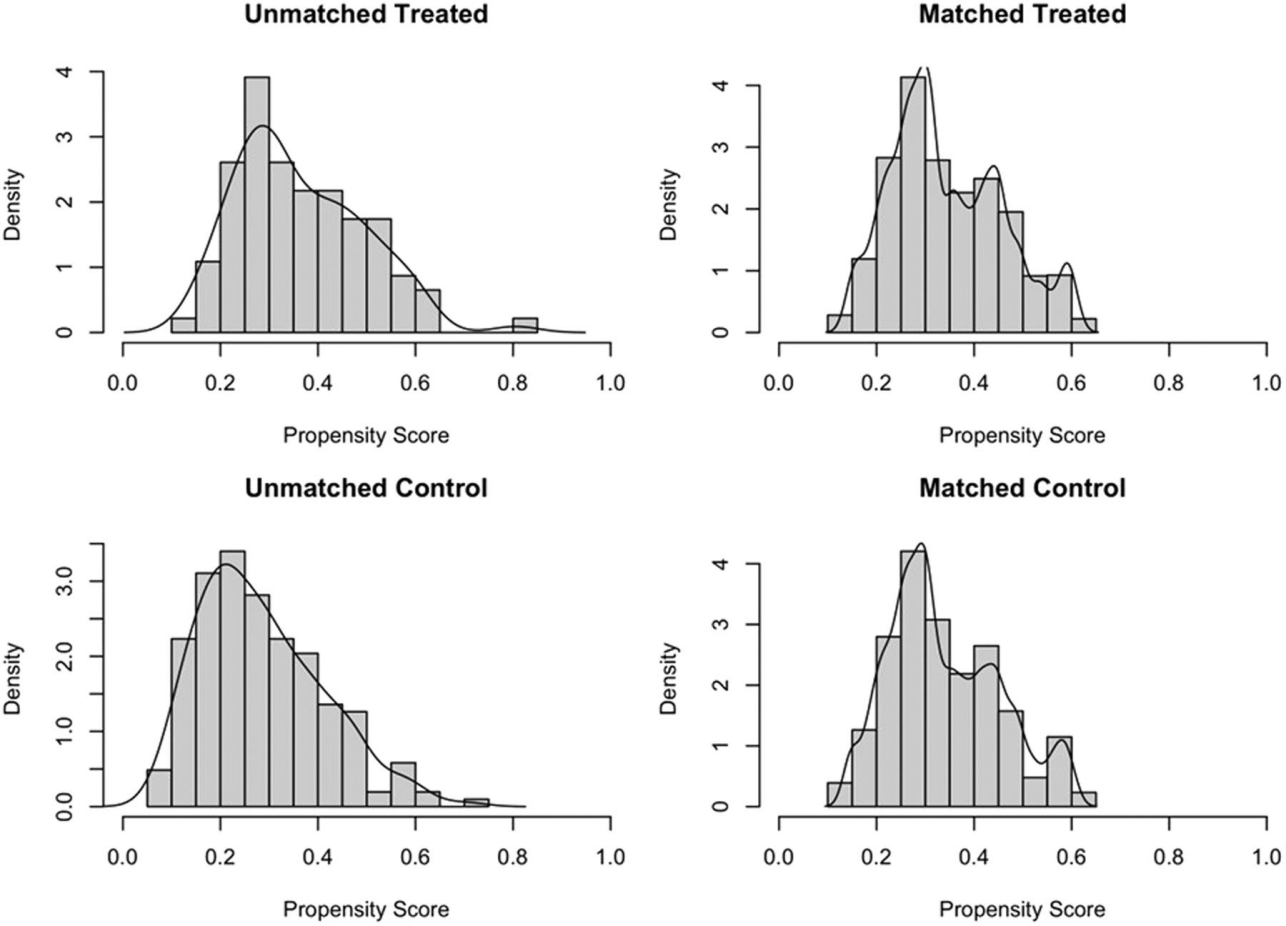
Propensity score distribution before and after matching. Density plots showing the distribution of propensity scores in the unmatched and matched cohorts. The distributions after matching indicate improved balance between the cases and control. The x‐axis represents the propensity score (probability of treatment assignment), and the y‐axis shows the density of observations.

After propensity‐score matching (Figure 1), 71 children were excluded. Therefore, a total of 227 children were included in the final analysis, 85 (37.5%) cases and 142 (62.5%) controls. The covariates included in the propensity score model, along with the standardized mean differences for each covariate before and after matching, are shown in Supporting Information: Figure 1.

The characteristics of the matched cohort are reported in Table 1. No differences emerged between cases and controls in terms of rate of ASC at the diagnosis at the index date and severity at anti‐TNF start.

**Table 1:** Baseline clinical and demographic characteristics of the population after propensity score matching analysis (matched cohort).^a^

	5‐ASA *N* = 142 (62.5%)	5‐ASA stop *N* = 85 (37.5%)	*p*‐value
Gender F, *n* (%)	82 (57.8)	53 (62.4)	0.5
Age (years) at the diagnosis, median (IQR)	12.5 (12–14.25)	11.9 (9.4–14)	0.6
UC disease extension at the diagnosis, *n* (%)			
E1	6 (4)	9 (11)	0.09
E2	24 (17)	20 (23)	0.22
E3	27 (19)	12 (14)	0.37
E4	85 (60)	44 (52)	0.21
ASC at the diagnosis, *n* (%)	45 (32)	30 (35)	0.66
Therapy prior to anti‐TNF, *n* (%)			
Systemic CS (> 90 days before anti‐TNF‐α)	84 (52)	43 (50.6)	0.21
CS recent (< 90 days before anti‐TNF‐α)	125 (88)	74 (87)	0.83
Immunomodulators, *n* (%)	86 (60.6)	56 (66)	0.4
5‐ASA	132 (93)	78 (82)	0.79
Sulfasalzine	10 (7)	7 (8)	
Anti‐TNF type, *n* (%)			
IFX	136 (95.8)	81 (95.3)	1
ADA	6 (4.2)	4 (4.7)	
Age (years) at anti‐TNF start, median (IQR)	14 (11.8–15.3)	13.2 (10–16)	0.32
Interval (months) from diagnosis and anti‐TNF start, median (IQR)	10 (3–25)	8 (4–23)	0.41
Standard dose	88 (62)	52 (61.2)	1
Accellerated induction regimen, *n* (%)	36 (25.4)	21 (24.7)	1
Therapeutic Drug Monitoring, *n* (%)	60 (42.3)	28 (32.9)	0.2
Immunomodulators, *n* (%)	77 (54.2)	48 (56.5)	0.78
UC disease extension at anti‐TNF start, *n* (%)			
E1	2 (1.4)	4 (4.7)	0.23
E2	21 (14.8)	14 (16.5)	0.84
E3	23 (16.2)	12 (14.1)	0.74
E4	96 (67.6)	55 (64.7)	0.66
ASC at anti‐TNF start, *n* (%)	34 (24)	20 (23.5)	1
Endoscopic evaluation at anti‐TNF start, *n* (%)	107 (75.4)	50 (59)	0.01
MAYO score at anti‐TNF start, median (IQR)	2 (2–3)	2 (2–3)	0.83
UCEIS at anti‐TNF start, median (IQR)	4 (3–6)	4 (3.5–6)	0.94
PUCAI at anti‐TNF start, median (IQR)	45 (35–65)	45 (30–60)	0.36
Laboratory values at anti‐TNF start, median (IQR)			
Hemoglobin (g/dL)	11.4 (10–11.9)	11.3 (8.6–12)	0.13
ESR (mm/h)	40 (23.5–45.5)	29 (18–42)	0.05
CRP (mg/L)	15.5 (5–35)	8 (5–30)	0.34
Albumin (g/L)	41 (35–44)	43 (37–46)	0.09
Fecal calprotectin (μg/g)	583 (374–1365)	433 (204–1358)	0.26
Clinical remission (PUCAI < = 10) at w 14, *n* (%)	94 (66.2)	52 (61.2)	0.4

Abbreviations: 5‐ASA, 5‐aminosalicylic acid; ADA, adalimumab; anti‐TNF, anti tumor necrosis factor; ASC, acute severe colitis; CRP, C‐reactive protein; ESR, erythrocyte sedimentation rate; IFX, infliximab; IQR, interquartile range; MAYO, Mayo Clinic Score; PUCAI, Pediatric Ulcerative Colitis Activity Index; UC, ulcerative colitis; UCEIS, Ulcerative Colitis Endoscopic Index of Severity.

a All data refers to the index date (start of anti‐TNF‐α), unless otherwise specified.

### 3.1 Risk of major adverse outcomes in cases and controls in the matched cohort

Differences between cases and controls were observed only at 6‐month follow up, when children discontinuing 5‐ASA resulted at higher risk of CS courses (OR 2.3, 95% CI 1.3–4, *p* = 0.003), hospitalizations (OR 2.1, 95% CI 1.03–4.2, *p* = 0.03), and acute severe colitis (OR 3.1, 95% CI 1.2–8, *p* = 0.02) (Supporting Information: Table 2). No other difference was observed for all the other outcomes evaluated at any time point.

**Figure 2:**
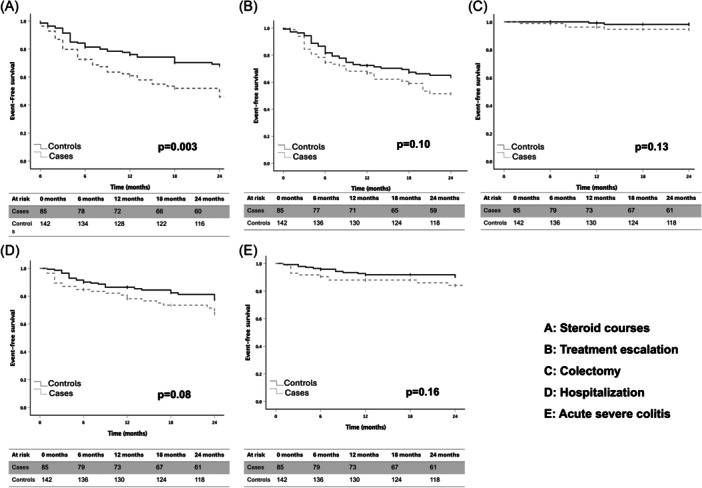
Kaplan–Meier survival analysis of all the outcomes. (A) Clinical relapses; (B) treatment escalation; (C) hospitalization; (D) steroid course; (E) complications.

The risk of steroid courses over time was higher in cases compared to the controls at the Kaplan–Meier survival analysis (Figure 2A *p* = 0.003), and a trend towards a higher risk of hospitalizations was observed (*p* = 0.08, Figure 2D). No significant differences were observed for the remaining outcomes evaluated between the two groups (Figure 2B,C,E).

### 3.2 Primary analysis (propensity score‐matched cohort)

Univariate and multivariate Cox regression analyses in the matched cohort is reported in Table 2. Discontinuation of 5‐ASA therapy at anti‐TNF initiation was independently associated with a significantly increased risk of requiring subsequent steroid courses (HR 1.95, 95% CI 1.2–3.0; *p* = 0.003). The association between 5‐ASA discontinuation and acute severe colitis demonstrated a strong univariate relationship (HR 1.8, 95% CI 1.2–2.7; *p* = 0.02), which attenuated to nonsignificance after multivariate adjustment (HR 1.6, 95% CI 0.7–3.7; *p* = 0.20). For hospitalization risk, a trend towards a significant association (HR 1.6, 95% CI 0.9–2.8; *p* = 0.08) was observed, while 5‐ASA discontinuation was not associated with increased risk of treatment escalation.

**Table 2:** Univariate and multivariate Cox regression analysis of factors associated with the risk of unfavorable outcomes following anti‐TNF initiation in the propensity score matched cohort.

Courses of steroids	HR (95% CI)	*p*‐value	HR (95% CI)	*p*‐value
5‐ASA discontinuation	1.9 (1.2–2.9)	0.003	1.95 (1.2–3.0)	0.003
ASC at the diagnosis	1.7 (1.1–2.6)	0.01	1.4 (0.9–2.2)	0.11
Gender F	0.92 (0.59–1.4)	0.72		
Age at the diagnosis	0.99 (0.99–1.003)	0.36		
Age at anti‐TNF start	0.99 (0.98–0.99)	0.01		
Interval from the diagnosis and anti‐TNF start (months)	0.98 (0.97–0.99)	0.03		
PUCAI at anti‐TNF start	0.99 (0.98–1.01)	0.83		
E3/E4	0.97 (0.56–1.6)	0.94	1 (0.5–1.7)	0.99
Previous CS ( > 90 days before anti‐TNF start)	0.76 (0.49–1.18)	0.23		
Previous CS ( < 90 days before anti‐TNF start)	1.77 (0.95–3.28)	0.06		
Immunomodulation at anti‐TNF start	0.87 (0.56–1.34)	0.54	0.6 (0.4–1.03)	0.07
IFX standard dose	0.83 (0.53–1.29)	0.41		
Accellerated induction regimen for IFX	1.2 (0.76–2.04)	0.36	1.0 (0.6–1.7)	0.82
TDM	1.5 (0.98–2.3)	0.059		
MAYO endoscopic subscore	0.81 (0.57–1.1)	0.26		
Albumin	1.04 (1.002–1.08)	0.03		
CRP	0.99 (0.97–1.009)	0.40		
ESR	0.99 (0.98–1.007)	0.62		
FC	1 (1–1)	0.10		
W14 clinical remission	0.47 (0.30–0.72)	<0.001	0.52 (0.3–0.8)	0.004
Acute severe colitis				
5‐ASA discontinuation	2.2 (1.1–4.3)	0.02	1.6 (0.7–3.7)	0.20
ASC at the diagnosis	1.7 (0.7–3.8)	0.17	1.5 (0.7–3.5)	0.26
Gender F	1.5 (0.6–3.4)	0.29		
Age at the diagnosis	0.99 (0.9–1.0)	0.28		
Age at anti‐TNF start	0.99 (0.98–1.0)	0.52		
Interval from the diagnosis and anti‐TNF start (months)	1.0 (0.9–1.0)	0.52		
PUCAI at anti‐TNF start	1.02 (1.0–1.04)	0.03		
E3/E4	0.69 (0.2–1.7)	0.43		
Previous CS (> 90 days before anti‐TNF start)	152 (0.6–3.4)	0.31		
Previous CS (< 90 days before anti‐TNF start)	2.3 (0.9–5.8)	0.07		
IM at anti‐TNF start	1.1 (0.5–2.6)	0.71		
IFX standard dose	0.6 (0.2–1.3)	0.24		
Accellerated induction regimen for IFX	1.0 (0.4–2.6)	0.92		
TDM	2.9 (1.3–6.8)	0.01		
MAYO endoscopic subscore	0.9 (0.4–2.0)	0.97		
Albumin	0.9 (0.9–1.06)	0.81		
CRP	0.9 (0.9–1.01)	0.13		
ESR	0.9 (0.9–1.0)	0.07		
FC	1 (1–1)	0.63		
W14 clinical remission	0.4 (0.1–0.9)	0.03	0.4 (0.2–1.0)	0.05
Hospitalization				
5‐ASA discontinuation	1.0 (0.9–2.7)	0.09	1.6 (0.9–2.8)	0.08
ASC at the diagnosis	1.3 (0.7–2.4)	0.26	1.1 (0.6–2.0)	0.70
Gender F	0.8 (0.4–1.4)	0.57		
Age at the diagnosis	0.9 (0.9–1.0)	0.55		
Age at anti‐TNF start	0.9 (0.9–1.0)	0.27		
Interval from the diagnosis and anti‐TNF start (months)	0.9 (0.9–1.0)	0.58		
PUCAI at anti‐TNF start	1.0 (0.9–1.0)	0.42		
E3/E4	1.2 (0.5–2.7)	0.53	1.3 (0.6–2.8)	0.49
Previous CS (> 90 days before anti‐TNF start)	0.7 (0.4–1.3)	0.32		
Previous CS (< 90 days before anti‐TNF start)	2.8 (1.2–6.1)	0.009		
IM at anti‐TNF start	1.03 (0.5–1.8)	0.90	0.7 (0.4–1.3)	0.29
IFX standard dose	1.0 (0.6–1.9)	0.79		
Accellerated induction regimen for IFX	1.1 (0.6–2.1)	0.70	1.0 (0.5–2.0)	0.86
TDM	2.0 (1.1–3.5)	0.01		
MAYO endoscopic subscore	0.6 (0.4–1.0)	0.07		
Albumin	1.0 (0.9–1.0)	0.65		
CRP	0.9 (0.9–1.0)	0.49		
ESR	0.9 (0.9–0.9)	0.03		
FC	1 (1–1)	0.62		
W14 clinical remission	0.4 (0.2–0.7)	0.004	0.4 (0.2–0.8)	0.008
Treatment escalation				
5‐ASA discontinuation	1.4 (0.9–2.2)	0.11	1.24 (0.8–1.9)	0.27
ASC at the diagnosis	1.7 (1.1–2.6)	0.01	1.4 (0.8–2.2)	0.15
Gender F	1.0 (0.6–1.5)	0.65		
Age at the diagnosis	1.0 (0.9–1.0)	0.64		
Age at anti‐TNF start	1 (0.99–1)	0.8		
Interval from the diagnosis and anti‐TNF start (months)	0.9 (0.9–1.0)	0.19		
PUCAI at anti‐TNF start	1 (0.9–1.0)	0.33		
E3/E4	1.4 (0.7–2.6)	0.22	1.5 (0.8–2.8)	0.17
Previous CS (> 90 days before anti‐TNF start)	0.7 (0.4–1.1)	0.16		
Previous CS (< 90 days before anti‐TNF start)	0.7 (0.6–2.0)	0.72		
IM at anti‐TNF start	0.8 (0.5–1.2)	0.40	0.6 (0.3–0.9)	0.02
IFX standard dose	1.1 (0.7–1.7)	0.65		
Accellerated induction regimen for IFX	1.1 (0.7–1.9)	0.50	0.9 (0.5–1.5)	0.70
TDM	1.0 (0.6–1.6)	0.86		
MAYO endoscopic subscore	0.9 (0.6–1.3)	0.92		
Albumin	1.0 (0.9–1.0)	0.34		
CRP	0.9 (0.9–1.0)	0.33		
ESR	0.9 (0.9–1.0)	0.41		
FC	1 (1–1)	0.002		
W14 clinical remission	0.2 (0.1–0.3)	<0.001	0.2 (0.1–0.3)	<0.001

Abbreviations: 5‐ASA, 5‐aminosalicylic acid; anti‐TNF, anti tumor necrosis factor; ASC, acute severe colitis; CI, confidence interval; CRP, C‐reactive protein; CS, corticosteroids; ESR, erythrocyte sedimentation rate; FC, fecal calprotectin; HR, hazard ratio; IFX, infliximab; IM, immunomodulation; MAYO, Mayo Clinic Score; PUCAI, Pediatric Ulcerative Colitis Activity Index; TDM, therapeutic drug monitoring.

Among the other relevant predictors, the most robust protective factor identified was achievement of clinical remission by Week 14, which was consistently associated, at the multivariate analysis, with reduced risks across all endpoints: steroid use (HR 0.52, 95% CI 0.3–0.8; *p* = 0.004), acute severe colitis (HR 0.4, 95% CI 0.2–1.0; *p* = 0.05), hospitalization (HR 0.4, 95% CI 0.2–0.8; *p* = 0.008), and treatment escalation (HR 0.2, 95% CI 0.1–0.3; *p* < 0.001). Concomitant immunomodulator use at anti‐TNF initiation demonstrated significant protection against treatment escalation (HR 0.6, 95% CI 0.3–0.9; *p* = 0.02) while recent steroid exposure (< 90 days before anti‐TNF initiation) emerged as a strong predictor of subsequent hospitalization (HR 2.8, 95% CI 1.2–6.1; *p* = 0.009) at a univariate analysis.

Baseline disease severity (S1) showed a significant univariate association with steroid use (HR 1.7, 95% CI 1.1–2.6; *p* = 0.01). Notably, demographic factors (gender and age), most inflammatory markers (CRP, ESR, and fecal calprotectin), and endoscopic activity scores did not show significant associations in multivariate models, nor did anti‐TNF dosing regimens (standard vs. accelerated induction).

### 3.3 Sensitivity analysis (unmatched cohort)

Univariate and multivariate Cox regression analyses in the unmatched cohort is reported in Supporting Information: Table 3. As in the matched analysis, 5‐ASA discontinuation remained strongly associated with steroid courses (HR = 1.98, 95% CI: 1.2–2.9, *p* < 0.001) and hospitalization (HR = 1.6, 95% CI 1–2.7, *p* = 0.04). Week 14 clinical remission was consistently protective across all endpoints (steroid use: HR = 0.4, 0.3–0.7, *p* < 0.001; treatment escalation: HR = 0.2, 0.14–0.3, *p* < 0.001; hospitalization: HR 0.4, 95% CI 0.2–0.68, *p* < 0.001; acute severe colitis: HR 0.4, 95% CI 0.2–0.8, *p* = 0.01). Disease severity (S1) showed stronger associations in the unmatched cohort for the subsequent risk of acute severe colitis (HR = 2.03, 1.01–4.1, *p* = 0.046 vs. non‐significant in matched), suggesting residual confounding in the primary analysis. Immunomodulator use demonstrated more robust protection against treatment escalation in the unmatched cohort (HR = 0.5, 0.4–0.89, *p* = 0.01 vs. HR = 0.6 in matched).

## 4 DISCUSSION

The introduction of anti‐TNF agents has significantly changed the treatment landscape for IBD, leading to improved outcomes for many patients.^13^ However, treatment efficacy is still limited, with approximately 60%–70% of patients responding positively. A substantial number of patients either do not respond initially (primary nonresponse) or experience a decline in response over time (secondary loss of response).^14^ This challenge highlights the need for better treatment strategies, particularly in pediatric patients, as anti‐TNF agents are currently the only biologic option approved for managing IBD in this population. The potential benefits of continuing 5‐ASA, which is commonly the first‐line therapy for UC in children,^2^ have not been thoroughly investigated in the context of starting biological treatment. Our study addresses this gap by showing that discontinuing 5‐ASA at anti‐TNF initiation is associated with a significantly higher hazard of subsequent steroid courses (log‐rank *p* = 0.003; adjusted HR 1.95), with the between‐group differences mainly evident within the first 6 months; accordingly, fixed timepoint analyses showed a statistically significant increase in the odds of adverse outcomes at the 6‐month follow‐up (including hospitalizations and acute severe colitis), with no significant differences detected at 12, 18, or 24 months, and only a non‐significant trend toward higher hospitalization risk over time. Cox regression analysis further substantiated these findings, demonstrating that achieving early clinical remission (by Week 14) significantly reduced the risk of adverse outcomes by 60%–80% (*p* ≤ 0.01). The robustness of these findings was evident in both unmatched and propensity score‐matched analyses.

In the matched cohort, which accounted for baseline confounders, recent steroid use (within 90 days) emerged as a strong predictor of hospitalization, while concurrent immunomodulator use was associated with a lower risk of treatment escalation. In contrast, the unmatched cohort exhibited variations in effect size due to residual confounding.

Our results differ from those of adult‐based studies, which have found that discontinuing 5‐ASA at the initiation of anti‐TNF therapy is not associated with worse clinical outcomes. A nationwide study conducted in Korea from 2007 to 2020 reported no increased risk of corticosteroid use, hospitalization, or surgery after the withdrawal of 5‐ASA.^15^ Similarly, a retrospective analysis of 121 adults with moderate‐to‐severe UC treated with infliximab showed no significant differences in clinical or endoscopic remission, hospitalization rates, rescue therapy, or colectomy between patients who did or did not receive mesalamine.^16^ A meta‐analysis by Singh et al. further supported the lack of additional benefits from mesalamine regarding remission, response, or mucosal healing.^17^ The absence of benefit was also noted in patients starting anti‐metabolite therapy.^6^ Furthermore, Ungaro et al. analyzed data from over 3500 patients in the United States and Denmark and found no increased risk of adverse outcomes following mesalamine discontinuation after the initiation of anti‐TNF therapy.^18^ Based on this evidence, the American Gastroenterological Association recommends discontinuing mesalamine in patients with moderate‐to‐severe UC who are starting biologics or small molecules and achieve remission.^19^ However, this recommendation is based on low‐quality evidence, and pediatric guidelines do not offer a similar directive.^2^ Additionally, the potential chemopreventive effect of 5‐ASA remains debated. Evidence from pre‐biologic era observational studies and meta‐analyses suggests an association between 5‐ASA exposure and a reduced risk of colorectal neoplasia in UC, with a signal that may be stronger at higher maintenance doses.^20^, ^21^, ^22^ Proposed mechanisms include both anti‐inflammatory effects and direct molecular actions on carcinogenesis pathways.^23^ However, these data are largely derived from nonrandomized cohorts and may be influenced by confounding (including inflammation severity and treatment era). In the biologic era, the independent additive benefit of 5‐ASA is increasingly uncertain: Nishida et al. found no measurable colorectal cancer–preventive effect of concomitant 5‐ASA among TNF inhibitor‐treated UC patients (adjusted HR 1.25, 95% CI 0.76–2.04).^24^ Contemporary reviews emphasize that durable control of inflammation, often achieved with biologics, likely drives colorectal cancer risk reduction, making any incremental protection from 5‐ASA unclear.^22^ This question is particularly relevant in pediatric‐onset UC, given the long disease duration and higher lifetime risk of colorectal cancer, supported by northern European population‐based data showing increased CRC risk in childhood‐onset IBD, especially extensive UC.^25^, ^26^ Therefore, the key preventive strategy should remain sustained control of intestinal inflammation (with appropriate surveillance), rather than continuation of 5‐ASA solely for putative chemoprevention in children in deep remission on biologics.

Overall, the data remains inconclusive, highlighting ongoing uncertainty^27^ and the lack of RCTs, particularly in the pediatric context. Although not designed to directly address the same question as our study, a recent population‐based analysis using the Epi‐IIRN Israeli cohort explored the potential impact of a no‐maintenance therapy approach and confirmed the specificities of the pediatric disease.^28^ In fact, while mesalamine withdrawal in newly diagnosed adults did not influence long‐term outcomes, pediatric patients—who often present with more extensive and severe disease—did not show comparable findings, indicating the need of continued maintenance therapy.^28^, ^29^, ^30^ To our knowledge, our study is the first to specifically investigate whether discontinuing 5‐ASA in children initiating biologic therapy affects outcomes, thus providing a foundation for more structured clinical trials.

Additional noteworthy findings emerged from our analysis, including the impact of early clinical remission on the likelihood of unfavorable outcomes. These results support the “earlier the better” approach, reinforcing the treat‐to‐target strategy, where an early clinical response serves as a critical short‐term treatment milestone. Multiple studies highlight the prognostic significance of early response assessment in UC across a range of biologic treatments.^31^, ^32^, ^33^

We acknowledge several limitations of our study, including the relatively small sample size and its retrospective design. However, the use of propensity‐score matching helps reduce bias resulting from the lack of randomization and from potential confounding by indication, including differences in disease severity at the index date (e.g., ASC)^34^ that could have influenced treatment decisions. Nonetheless, residual confounding cannot be completely excluded, particularly because we did not systematically collect cumulative systemic corticosteroid exposure before anti‐TNF initiation.^35^ Additionally, while our study underscores the potential clinical benefits of continuing mesalamine therapy, it does not address the social, economic, and psychological burdens associated with long‐term oral medication, especially in adolescents and young adults. In this regard, an alternative strategy worth investigating is dose reduction, which could provide significant cost savings and alleviate the burden of taking multiple pills.^27^

## 5 CONCLUSIONS

Discontinuing mesalamine at the time of anti‐TNF initiation in pediatric UC is associated with an increased risk of early disease exacerbation and medical interventions, mainly a higher hazard of subsequent corticosteroid courses, mostly within the first 6 months, with no significant differences at later follow‐up timepoints. Treatment decisions should be individualized, taking into account patient preferences, disease characteristics, early biologic response, adherence to treatment, and cost‐benefit analyses.^36^, ^37^ Given mesalamine's favorable safety profile, affordability, and potential to reduce early relapses and steroid exposure, its continuation in pediatric patients when initiating anti‐TNF therapy appears to be a prudent approach; based on our findings, a pragmatic strategy could be to maintain 5‐ASA for the first 6 months after anti‐TNF initiation to help consolidate the therapeutic benefit of biologic therapy, before reassessing the need for ongoing 5‐ASA thereafter. Our findings provide a basis for future prospective studies aimed at further defining the role of mesalamine as complementary in the treatment armamentarium of pediatric UC.

## CONFLICT OF INTEREST STATEMENT

The authors declare no conflicts of interest.

## Supporting information


**Supplementary figure 1 (SDC2):** Covariate balance before and after matching (standardized mean differences). Jitter plot of standardized mean differences for each baseline covariate comparing exposure groups, shown before matching (white dots) and after propensity score matching (black dots). *PSM: propensity score matching; CS: corticosteroid; ASC: acute severe colitis; TNF: tumor necrosis factor; PUCAI: pediatric ulcerative colitis activity index; IM: immunomodulator*.

supplementary table 1_mod.

supplementary table 2.

Supplementary table 3_mod.
