# Supplementary figures and images for: Impact of 5‐Aminosalicylic acid discontinuation in children with ulcerative colitis on biologic therapy: A propensity score‐matched study
Source: J Pediatr Gastroenterol Nutr. 2026 Mar 26;83(1):96–107. doi: 10.1002/jpn3.70415 (PMC13342762; doi:10.1002/jpn3.70415)

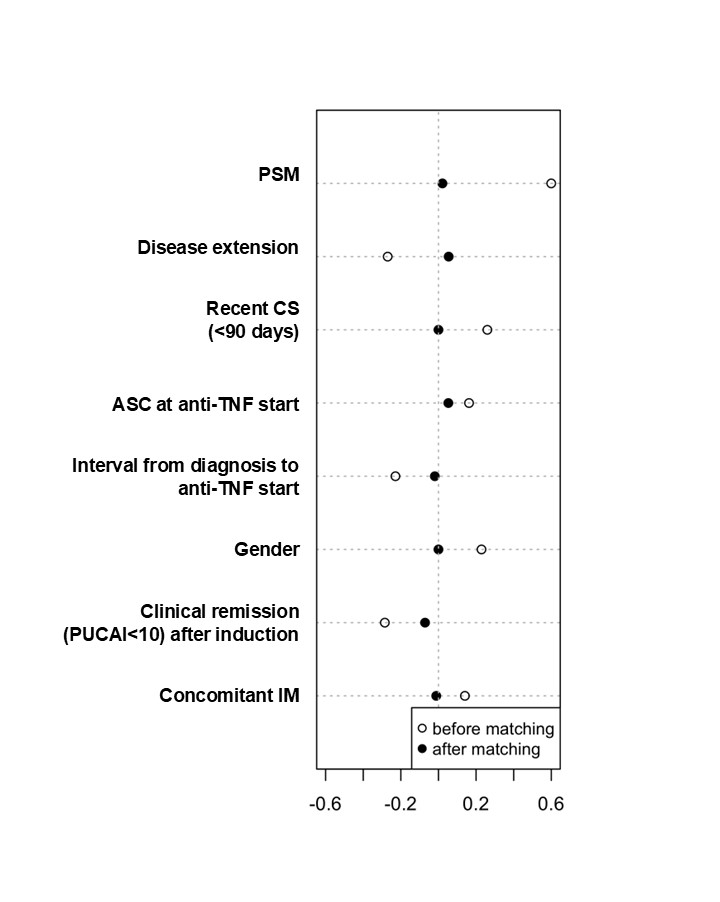

Supplement: Supplementary file 1 — Supplementary figure 1 (SDC2): Covariate balance before and after matching (standardized mean differences). Jitter plot of standardized mean differences for each baseline covariate comparing exposure groups, shown before matching (white dots) and after propensity score matching (black dots). PSM: propensity score matching; CS: corticosteroid; ASC: acute severe colitis; TNF: tumor necrosis factor; PUCAI: pediatric ulcerative colitis activity index; IM: immunomodulator. [file JPN3-83-96-s001.jpg]
