# Supplementary material for: Impact of 5‐Aminosalicylic acid discontinuation in children with ulcerative colitis on biologic therapy: A propensity score‐matched study
Source: J Pediatr Gastroenterol Nutr. 2026 Mar 26;83(1):96–107. doi: 10.1002/jpn3.70415 (PMC13342762; doi:10.1002/jpn3.70415)
Supplement: Supplementary file 2 — supplementary table 1_mod. [file JPN3-83-96-s002.docx]

| **Supplementary table 1.** Baseline clinical and demographic characteristics of the population before propensity score matching analysis (unmatched cohort)*. | | | |
| --- | --- | --- | --- |
|  | **5-ASA**  **N=206 (69%)** | **5-ASA stop**  **N= 92 (31%)** | **p** |
| **Gender F, n (%)** | 107 (52) | 58 (63) | 0.07 |
| **Age (years) at the diagnosis, median (IQR)** | 12 (8.3-14) | 12 (9.4-14.2) | 0.31 |
| **UC disease extension at the diagnosis, n (%)**  E1  E2  E3  E4 | 12 (5.8)  34 (16.5)  35 (17)  125 (60.7) | 9 (10)  25 (27.2)  12 (13)  46 (50) | 0.21  0.04  0.48  0.09 |
| **ASC at the diagnosis, n (%)** | 51 (24.8) | 33 (35.9) | 0.05 |
| **Therapy prior to anti-TNF, n (%)**  Systemic CS (>90 gg before anti-TNFα)  CS recent (<90 gg before anti-TNFα)  Immunomodulators, n (%) | 118 (57.3)  161 (78.2)  117 (56.8) | 46 (50)  80 (87)  61 (66.3) | 0.25  0.08  0.12 |
| **5-ASA**  **Sulfasalazine** | 194 (94)  12 (6) | 77 (90)  9 (10) | 0.21 |
| **Anti-TNF type, n (%)**  IFX  ADA | 198 (96.1)  8 (3.9) | 85 (92.4)  7 (7.6) | 0.24 |
| **Age (years) at anti-TNF start, median (IQR)** | 14 (11.2-15.3) | 13.5 (10.4-16) | 0.32 |
| **Interval (months) from diagnosis and anti-TNF start, median (IQR)** | 10 (3-29) | 8 (3-23) | 0.07 |
| **Therapeutic Drug Monitoring, n (%)** | 91 (44.2) | 30 (32.6) | 0.07 |
| **Immunomodulators, n (%)** | 102 (49.5) | 52 (56.5) | 0.32 |
| **UC disease extension at anti-TNF start, n (%)**  E1  E2  E3  E4 | 4 (1.9)  26 (12.6)  37 (18)  139 (67.5) | 4 (4.4)  18 (19.6)  12 (13)  58 (63) | 0.25  0.15  0.31  0.54 |
| **ASC at anti-TNF start, n (%)** | 41 (19.8) | 25 (27.2) | 0.17 |
| **Endoscopic evaluation at anti-TNF start, n (%)** | 107 (75.4) | 50 (58.8) | 0.01 |
| **MAYO score at anti-TNF start, median (IQR)** | 2 (2-3) | 2 (2-3) | 0.58 |
| **UCEIS at anti-TNF start, median (IQR)** | 4 (3-5) | 4 (4-6) | 0.53 |
| **PUCAI at anti-TNF start, median (IQR)** | 40 (30-55) | 45 (30-60) | 0.62 |
| **Laboratory values at anti-TNF start, median (IQR)**  Hemoglobin (gr/dL)  ESR (mm/h)  CRP (mg/L)  Albumin (g/L)  Fecal calprotectin (mcg/gr) | 11.9 (11.4-12.8)  36 (21-55)  7 (1.5-19.2)  40 (36-44)  738 (383-1416) | 12 (11-12.9)  29.5 (18-50)  4.2 (1.2-11)  43 (37-46)  437 (200-1910) | 0.10  0.25  0.98  0.08  0.43 |
| **Clinical remission (PUCAI <=10) w 14, n (%)** | 150 (72.8) | 54 (58.7) | 0.02 |
| ** All data refers to the index date (start of anti-**TNFα), unless otherwise specified. 5-ASA: 5-aminosalicylic acid; IQR: interquartile range; UC: ulcerative colitis; anti-TNF: anti tumor necrosis factor; PUCAI: pediatric ulcerative colitis activity index; IFX: infliximab; ADA: adalimumab; ASC: acute severe colitis; UCEIS: ulcerative colitis endoscopic index of severity; CRP: C-reactive protein; ESR: erythrocyte sedimentation rate* | | | |
