# Supplementary material for: Impact of 5‐Aminosalicylic acid discontinuation in children with ulcerative colitis on biologic therapy: A propensity score‐matched study
Source: J Pediatr Gastroenterol Nutr. 2026 Mar 26;83(1):96–107. doi: 10.1002/jpn3.70415 (PMC13342762; doi:10.1002/jpn3.70415)
Supplement: Supplementary file 3 — supplementary table 2. [file JPN3-83-96-s004.docx]

| ***Supplementary table 2.*** *Risk of adverse outcomes in cases and controls at 6, 12 and 24 months* | | | | |
| --- | --- | --- | --- | --- |
| **Outcome** | **5-ASA**  **N=142** | **5-ASA STOP**  **N=85** | **Chi-square p value** | **OR (95% CIs)** |
| **6 months**  Treatment escalation, n (%)  CS courses, n (%)  Hospitalization, n (%)  Clinical relapses, n (%)  Colectomy, n (%)  Acute severe colitis, n (%)  Mucosal healing, n (%) | 31 (22)  39 (27)  17 (12)  49 (34.5)  0 (-)  7 (5)  33 (23) | 25 (29)  40 (47)  19 (22)  31 (36)  1 (1)  12 (14)  26 (30.5) | 0.34  0.003  0.03  0.77  0.37  0.02  0.2 | 0.7 (0.3-1.3)  2.3 (1.3-4)  2.1 (1.0-4.2)  1.0 (0.6-1.9)  Inf (0.1-inf)  3.1 (1.2-8)  1.4 (0.8-2.6) |
| **12 months**  Treatment escalation, n (%)  CS courses, n (%)  Hospitalization, n (%)  Clinical relapses, n (%)  Colectomy, n (%)  Acute severe colitis  Mucosal healing, n (%) | 21 (15)  24 (17)  10 (7)  25 (18)  1 (1)  4 (3)  43 (30) | 16 (19)  20 (23.5)  10 (12)  15 (18)  3 (3.5)  4 (5)  33 (39) | 0.46  0.22  0.2  0.99  0.14  0.48  0.19 | 1.3 (0.6-2.7)  1.5 (0.7-2.8)  1.7 (0.6-4.5)  1.0 (0.5-1.9)  5.1 (0.7-67.4)  1.6 (0.4-5.8)  1.4 (0.8-2.6) |
| **18 months**  Treatment escalation, n (%)  CS courses, n (%)  Hospitalization, n (%)  Clinical relapses, n (%)  Colectomy, n (%)  Acute severe colitis  Mucosal healing, n (%) | 11 (8)  14 (10)  6 (4)  17 (12)  1 (1)  1 (1)  29 (20) | 6 (7)  9 (10.5)  6 (7)  9 (10.5)  0 (-)  0 (-)  27 (32) | 0.99  0.99  0.37  0.83  0.99  0.99  0.058 | 0.9 (0.3-2.5)  1.0 (0.4-2.6)  1.7 (0.5-5.6)  0.8 (0.3-1.9)  0.0 (0-15)  0.0 (0-15)  1.8 (1-3.2) |
| **24 months**  Treatment escalation, n (%)  CS courses, n (%)  Hospitalization, n (%)  Clinical relapses, n (%)  Colectomy, n (%)  Acute severe colitis, n (%)  Mucosal healing, n (%) | 6 (4)  10 (7)  4 (3)  10 (7)  0 (-)  1 (1)  34 (24) | 9 (10.5)  12 (14)  3 (3.5)  8 (9)  0 (-)  4 (5)  21 (25) | 0.09  0.1  0.99  0.6  -  0.06  0.99 | 2.6 (0.9-7.8)  2.7 (0.94-5.2)  1.2 (0.3-4.7)  1.3 (0.5-3.7)  -  6.9 (1.1-85.7)  1.04 (0.5-1.9) |
| *OR: odds ratio; CIs: confidence intervals; 5-ASA:* 5*-aminosalicylic acid (5-ASA); CS: corticosteroid* | | | | |
