# Supplementary material for: Impact of 5‐Aminosalicylic acid discontinuation in children with ulcerative colitis on biologic therapy: A propensity score‐matched study
Source: J Pediatr Gastroenterol Nutr. 2026 Mar 26;83(1):96–107. doi: 10.1002/jpn3.70415 (PMC13342762; doi:10.1002/jpn3.70415)
Supplement: Supplementary file 4 — Supplementary table 3_mod. [file JPN3-83-96-s003.docx]

| **Supplementary table 3:** Univariate and multivariate Cox regression analysis of factors associated with the risk of unfavorable outcomes following anti-TNFα initiation in the unmatched cohort. | | | | |
| --- | --- | --- | --- | --- |
| **Courses of steroids** | **HR (95% CI)** | **P** | **HR (95% CI)** | **p** |
|  |  |  |  |  |
| 5-ASA discontinuation | 2.1 (1.4-3.13) | <0.001 | 1.98 (1.2-2.9) | <0.001 |
| ASC at the diagnosis | 1.8 (1.2-2.7) | 0.002 | 1.4 (0.9-2.1) | 0.1 |
| Gender F | 0.7 (0.5-1.1) | 0.2 |  |  |
| Age at the diagnosis | 1 (0.9-1) | 0.8 |  |  |
| Age at anti-TNF start | 0.99 (0.99-1) | 0.03 |  |  |
| Interval from the diagnosis and anti-TNF start (months) | 0.98 (0.98-0.99) | 0.01 |  |  |
| PUCAI at anti-TNF start | 1 (0.9-1.01) | 0.3 |  |  |
| E3/E4 | 0.79 (0.49-1.2) | 0.33 | 0.9 (0.5-1.4) | 0.67 |
| Previous CS (>90 days before anti-TNF start) | 0.89 (0.6-1.3) | 0.5 |  |  |
| Previous CS (<90 days before anti-TNF start) | 1.5 (0.93-2.5) | 0.08 |  |  |
| IM at anti TNF start | 0.89 (0.6-1.3) | 0.55 | 0.6 (0.4-0.98) | 0.04 |
| IFX standard dose | 0.74 (0.5-1.09) | 0.13 |  |  |
| Accellerated induction regimen for IFX | 1.3 (0.89-2.1) | 0.14 | 1.1 (0.7-1.7) | 0.6 |
| TDM | 1.3 (0.9-2.04) | 0.09 |  |  |
| MAYO endoscopic subscore | 0.8 (0.6-1.2) | 0.46 |  |  |
| Albumin | 1.03 (0.99-1.03) | 0.1 |  |  |
| CRP | 0.99 (0.98-1) | 0.53 |  |  |
| ESR | 1 (0.99-1) | 0.96 |  |  |
| FC | 1 (1-1) | 0.26 |  |  |
| W14 clinical remission | 0.39 (0.2-0.57) | <0.001 | 0.4 (0.3-0.7) | <0.001 |
|  |  |  |  |  |
| **Acute severe colitis** | **HR (95% CI)** | **P** | **HR (95% CI)** | **p** |
| 5-ASA discontinuation | 2.2 (1.1-4.3) | 0.02 | 1.8 (0.9-3.3) | 0.7 |
| ASC at the diagnosis | 2.5 (1.3-5.1) | 0.006 | 2.0 (1.0-4.1) | 0.04 |
| Gender F | 0.9 (0.4-1.8) | 0.8 |  |  |
| Age at the diagnosis | 0.9 (0.9-1.0) | 0.61 |  |  |
| Age at anti-TNF start | 0.9 (0.9-1.0) | 0.79 |  |  |
| Interval from the diagnosis and anti-TNF start (months) | 1.0 (0.9-1.0) | 0.83 |  |  |
| PUCAI at anti-TNF start | 1.0 (1.0-1.0) | 0.007 |  |  |
| E3/E4 | 0.4 (0.2-1.0) | 0.06 |  |  |
| Previous CS (>90 days before anti-TNF start) | 1.2 (0.6-2.4) | 0.56 |  |  |
| Previous CS (<90 days before anti-TNF start) | 1.8 (0.7-4.0) | 0.15 |  |  |
| IM at anti TNF start | 1.1 (0.5-2.2) | 0.71 |  |  |
| IFX standard dose | 0.4 (0.2-0.9) | 0.035 |  |  |
| Accellerated induction regimen for IFX | 1.3 (0.6-2.9) | 0.44 |  |  |
| TDM | 2.2 (1.1-4.4) | 0.023 |  |  |
| MAYO | 1.08 (0.5-2.8) | 0.89 |  |  |
| Albumin | 0.9 (0.9-1.0) | 0.81 |  |  |
| CRP | 0.9 (0.9-1.0) | 0.08 |  |  |
| ESR | 0.9 (0.9-1.0) | 0.15 |  |  |
| FC | 1.0 (1.0-1.0) | 0.65 |  |  |
| W14 clinical remission | 0.33 (0.1-0.6) | 0.002 | 0.4 (0.2-0.8) | 0.01 |
|  |  |  |  |  |
| **Hospitalization** | **HR (95% CI)** | **P** | **HR (95% CI)** | **p** |
| 5-ASA discontinuation | 1.8 (1.1-2.9) | 0.013 | 1.6 (1-2.7) | 0.048 |
| ASC at the diagnosis | 1.8 (1.1-3) | 0.01 | 1.4 (0.8-2.3) | 0.19 |
| Gender F | 0.8 (0.5-1.3) | 0.4 |  |  |
| Age at the diagnosis | 1.0 (0.9-1.0) | 0.85 |  |  |
| Age at anti-TNF start | 0.9 (0.9-1.0) | 0.62 |  |  |
| Interval from the diagnosis and anti-TNFα start (months) | 0.9 (0.9-1) | 0.35 |  |  |
| PUCAI at anti-TNF start | 1 (0.9-1.02) | 0.08 |  |  |
| E3/E4 | 1.0 (0.5-1.9) | 0.9 | 1.1 (0.5-2.1) | 0.7 |
| Previous CS (>90 days before anti-TNF start) | 0.8 (0.5-1.3) | 0.5 |  |  |
| Previous CS (<90 days before anti-TNF start) | 1.5 (0.8-2.9) | 0.1 |  |  |
| IM at anti TNF start | 1.1 (0.7-1.8) | 0.55 | 0.8 (0.5-1.3) | 0.4 |
| IFX standard dose | 0.7 (0.4-1.2) | 0.3 |  |  |
| Accellerated induction regimen for IFX | 1.2 (0.7-2.1) | 0.4 | 1.0 (0.6-1.9) | 0.7 |
| TDM | 1.8 (1.1-3.0) | 0.01 |  |  |
| MAYO endoscopic subscore | 0.7 (0.5-1.1) | 0.18 |  |  |
| Albumin | 0.9 (0.9-1.0) | 0.8 |  |  |
| CRP | 1.0 (0.9-1.0) | 0.7 |  |  |
| ESR | 0.9 (0.9-1.0) | 0.7 |  |  |
| FC | 1.0 (1.0-1.0) | 0.62 |  |  |
| W14 clinical remission | 0.3 (0.2-0.5) | <0.001 | 0.4 (0.2-0.6) | <0.001 |
|  |  |  |  |  |
|  |  |  |  |  |
| **Treatment escalation** | **HR (95% CI)** | **P** | **HR (95% CI)** | **p** |
| 5-ASA discontinuation | 1.8 (1.1-2.9) | 0.013 | 1.1 (0.7-1.7) | 0.51 |
| ASC at the diagnosis | 1.9 (1.3-2.9) | <0.001 | 1.5 (0.9-2.3) | 0.06 |
| Gender F | 0.9 (0.6-1.3) | 0.61 |  |  |
| Age at the diagnosis | 1.0 (0.9-1.0) | 0.16 |  |  |
| Age at anti-TNF start | 1.0 (0.9-1.0) | 0.8 |  |  |
| Interval from the diagnosis and anti-TNF start (months) | 0.9 (0.9-1.0) | 0.03 |  |  |
| PUCAI at anti-TNF start | 1.0 (0.9-1.0) | 0.06 |  |  |
| E3/E4 | 1.1 (0.6-1.8) | 0.67 | 1.4 (0.8-2.3) | 0.21 |
| Previous CS (>90 days before anti-TNF start) | 0.8 (0.5-1.2) | 0.44 |  |  |
| Previous CS (<90 days before anti-TNF start) | 0.9 (0.5-1.5) | 0.83 |  |  |
| IM at anti TNF start | 0.8 (0.5-1.2) | 0.3 | 0.5 (0.4-0.8) | 0.01 |
| IFX standard dose | 1.0 (0.7-1.6) | 0.68 |  |  |
| Accellerated induction regimen for IFX | 1.2 (0.7-1.9) | 0.4 | 0.8 (0.5-1.4) | 0.58 |
| TDM | 1.1 (0.7-1.7) | 0.48 |  |  |
| MAYO endoscopic subscore | 1.0 (0.7-1.5) | 0.59 |  |  |
| Albumin | 1.0 (0.9-1.0) | 0.41 |  |  |
| CRP | 0.9 (0.9-1.0) | 0.14 |  |  |
| ESR | 1 (0.9-1.0) | 0.99 |  |  |
| FC | 1.0 (1.0-1.0) | 0.002 |  |  |
| W14 clinical remission | 0.2 (0.1-0.3) | <0.001 | 0.2 (0.1-0.3) | <0.001 |
| *HR: hazard ratio; CI: confidence interval; 5-ASA: 5-aminosalicylic acid; ASC: acute severe colitis; anti-TNF: anti tumor necrosis factor; CS: corticosteroids; IFX: infliximab; TDM: therapeutic drug monitoring; PUCAI: pediatric ulcerative colitis activity index; CRP: C-reactive protein; ESR: erythrocyte sedimentation rate; FC: fecal calprotectin.* | | | | |
